# Supplementary material for: Molecular basis of Mg2+ permeation through the human mitochondrial Mrs2 channel
Source: Nat Commun. 2023 Aug 5;14:4713. doi: 10.1038/s41467-023-40516-2 (PMC10404273; doi:10.1038/s41467-023-40516-2)
Supplement: Supplementary file 1 — Supplementary Information [file 41467_2023_40516_MOESM1_ESM.pdf]

Supplementary Information for

Molecular basis of Mg^{2+} permeation through the human mitochondrial Mrs2 channel

Ming Li, Yang Li, Yue Lu, Jianhui Li, Xuhang Lu, Yue Ren, Tianlei Wen, Yaojie Wang, Shenghai Chang, Xing Zhang, Xue Yang*, Yuequan Shen*

* Corresponding author.

Xue Yang (yangxue@nankai.edu.cn); Yuequan Shen (yshen@nankai.edu.cn)

This PDF file contains:

Supplementary Fig. 1-9

Supplementary Table 1-3

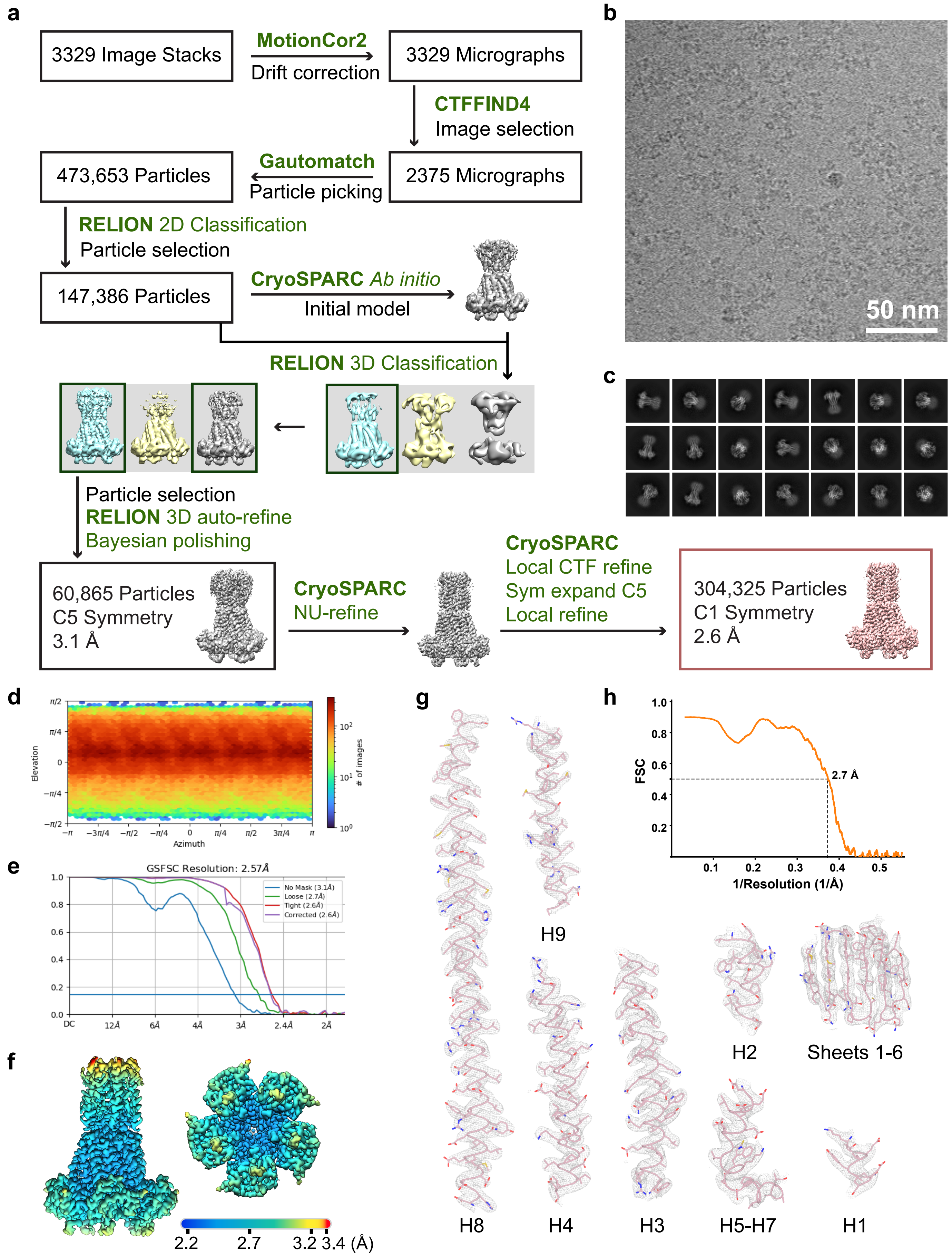

Supplementary Fig. 1 Structural determination of the hMrs2-Mg structure. **a** Workflow of image processing, 3D reconstruction and structure refinement of hMrs2-Mg. **b** Representative of 3,329 drift-corrected cryo-EM micrographs of hMrs2-Mg. **c** Representative 2D class averages of hMrs2-Mg. **d** Euler angle distribution of hMrs2-Mg in the final 3D reconstruction in cryoSPARC v3. **e** The gold standard FSC curve of hMrs2-Mg estimated by cryoSPARC v3. **f** Local resolution estimation from ResMap. **g** Representative cryo-EM density maps (gray mesh) superposed with hMrs2-Mg structure. Secondary structural elements of hMrs2-Mg are shown in a cartoon. **h** Cross-validated FSC curve between map and model.

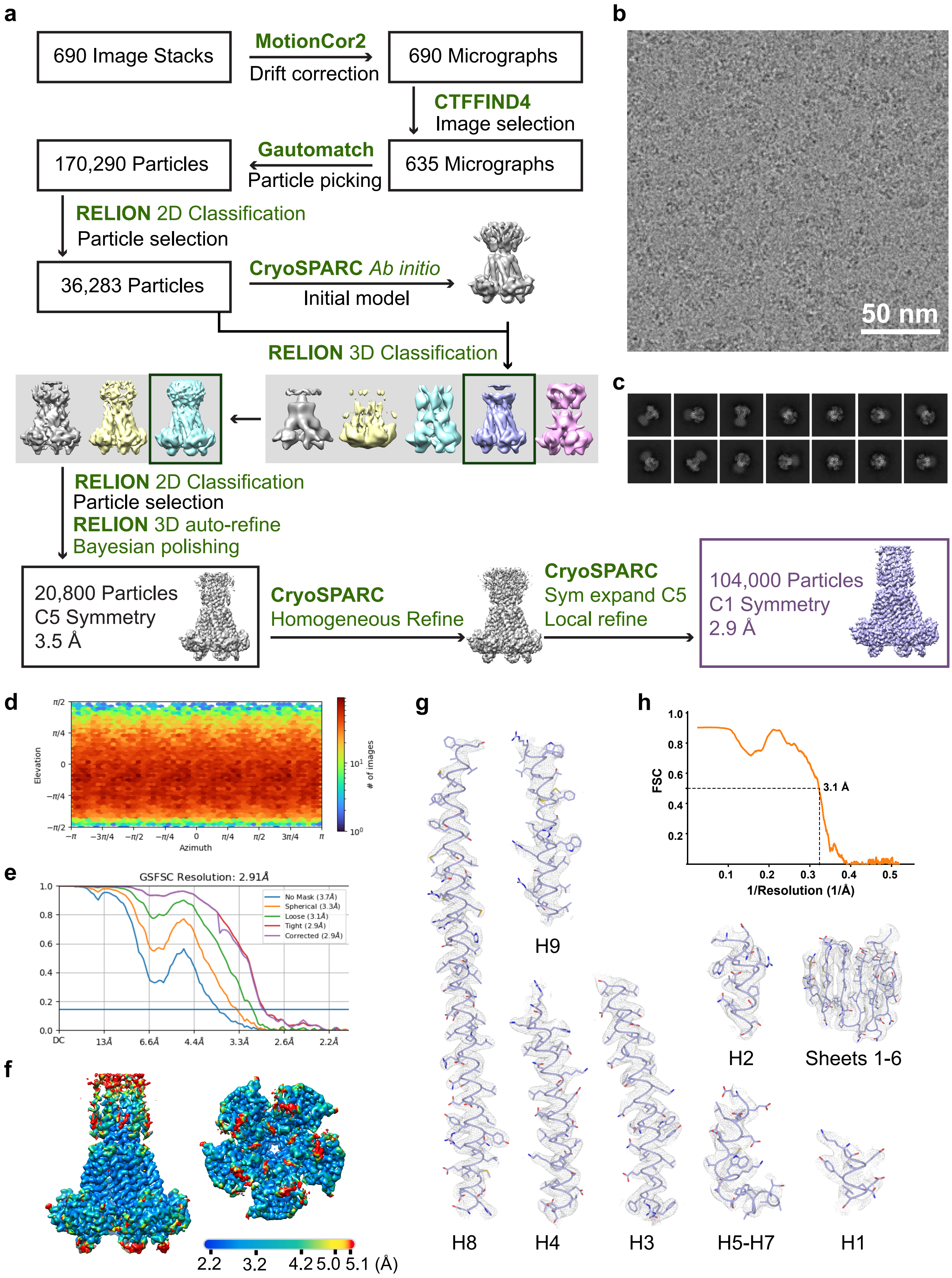

Supplementary Fig. 2 Structural determination of the hMrs2-rest structure. a Workflow of image processing, 3D reconstruction and structure refinement of hMrs2-rest. **b** Representative of 690 drift-corrected cryo-EM micrographs of hMrs2-rest. **c** Representative 2D class averages of hMrs2-rest. **d** Euler angle distribution of hMrs2-rest in the final 3D reconstruction in cryoSPARC v3. **e** The gold standard FSC curve of hMrs2-rest estimated by cryoSPARC v3. **f** Local resolution estimation from ResMap. **g** Representative cryo-EM density maps (gray mesh) superposed with hMrs2-rest structure. Secondary structural elements of hMrs2-rest are shown in a cartoon. **h** Cross-validated FSC curve between map and model.

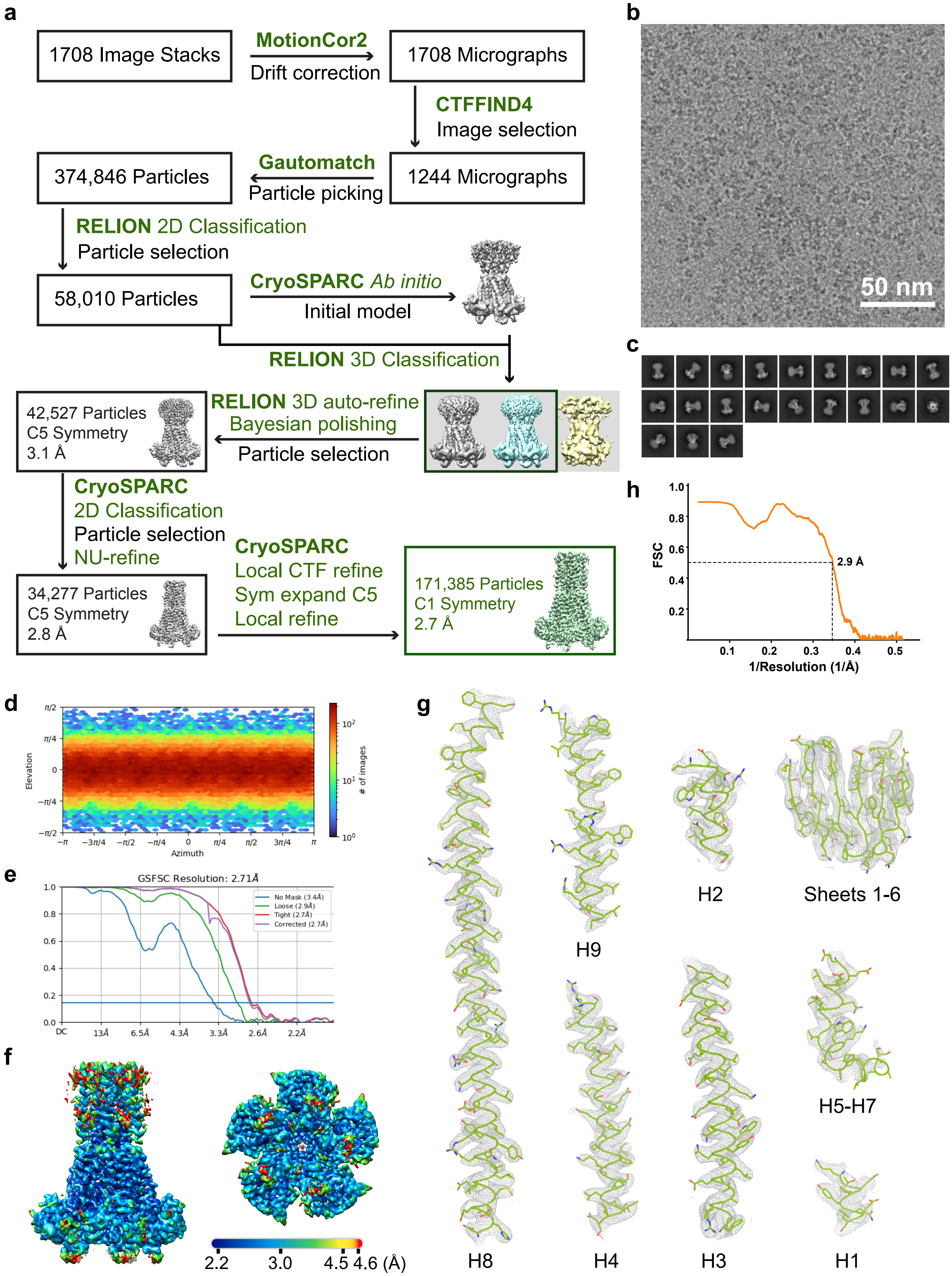

Supplementary Fig. 3 Structural determination of the hMrs2-highEDTA structure. a Workflow of image processing, 3D reconstruction and structure refinement of hMrs2-highEDTA. **b** Representative of 1,708 drift-corrected cryo-EM micrographs of hMrs2-highEDTA. **c** Representative 2D class averages of hMrs2-highEDTA. **d** Euler angle distribution of hMrs2-highEDTA in the final 3D reconstruction in cryoSPARC v3. **e** The gold standard FSC curve of hMrs2-highEDTA estimated by cryoSPARC v3. **f** Local resolution estimation from ResMap. **g** Representative cryo-EM density maps (gray mesh) superposed with hMrs2-rest structure. Secondary structural elements of hMrs2-rest are shown in a cartoon. **h** Cross-validated FSC curve between map and model.

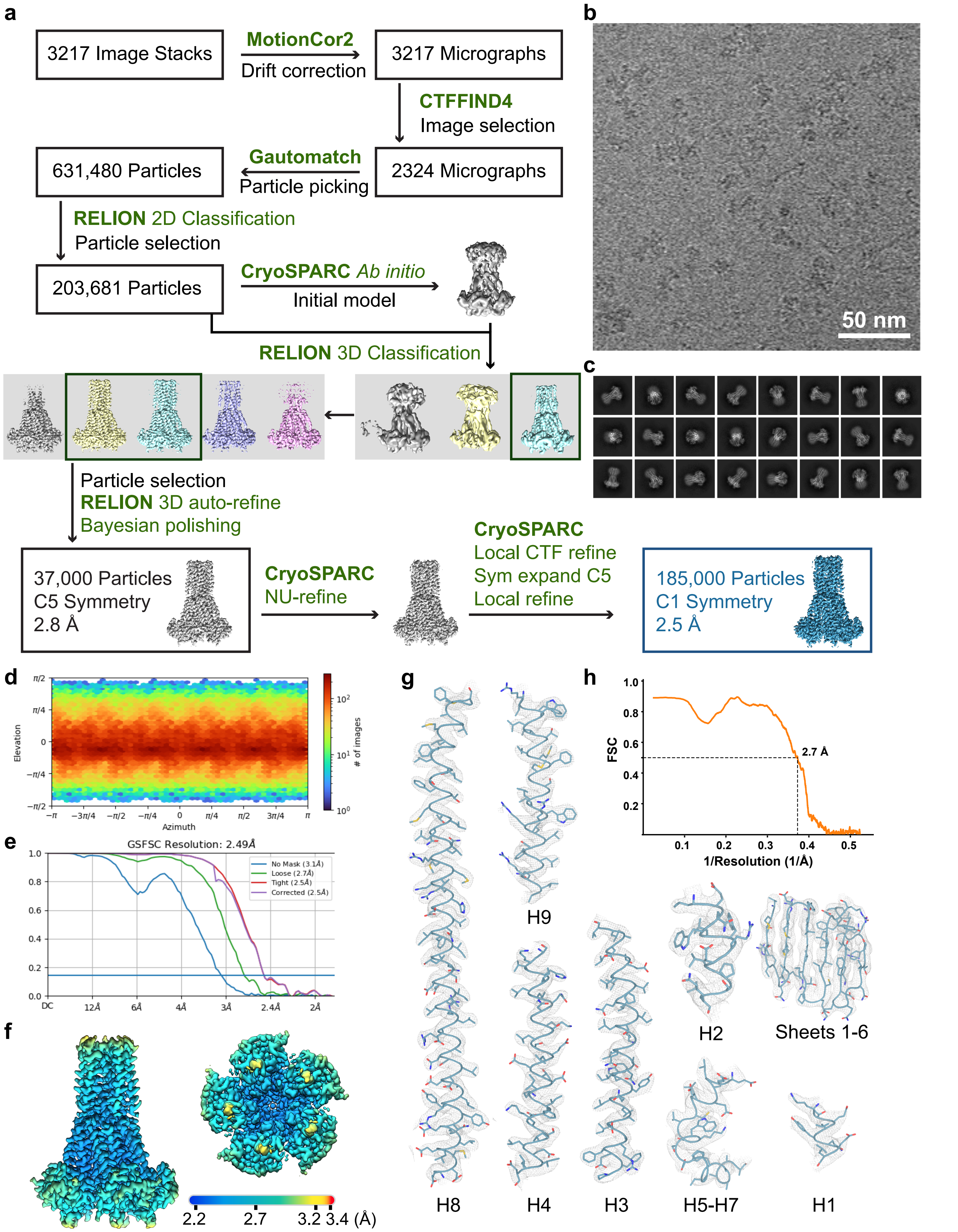

Supplementary Fig. 4 Structural determination of the hMrs2-lowEDTA structure. a workflow of image processing, 3D reconstruction and structure refinement of hMrs2-lowEDTA. **b** Representative of 3,217 drift-corrected cryo-EM micrograph of hMrs2-lowEDTA. **c** Representative 2D class averages of hMrs2-lowEDTA. **d** Euler angle distribution of hMrs2-lowEDTA in the final 3D reconstruction in cryoSPARC v3. **e** The gold standard FSC curve of hMrs2-lowEDTA estimated by cryoSPARC v3. **f** Local resolution estimation from ResMap. **g** Representative cryo-EM density maps (gray mesh) superposed with hMrs2-rest structure. Secondary structural elements of hMrs2-rest are shown in a cartoon. **h** Cross-validated FSC curve between map and model.

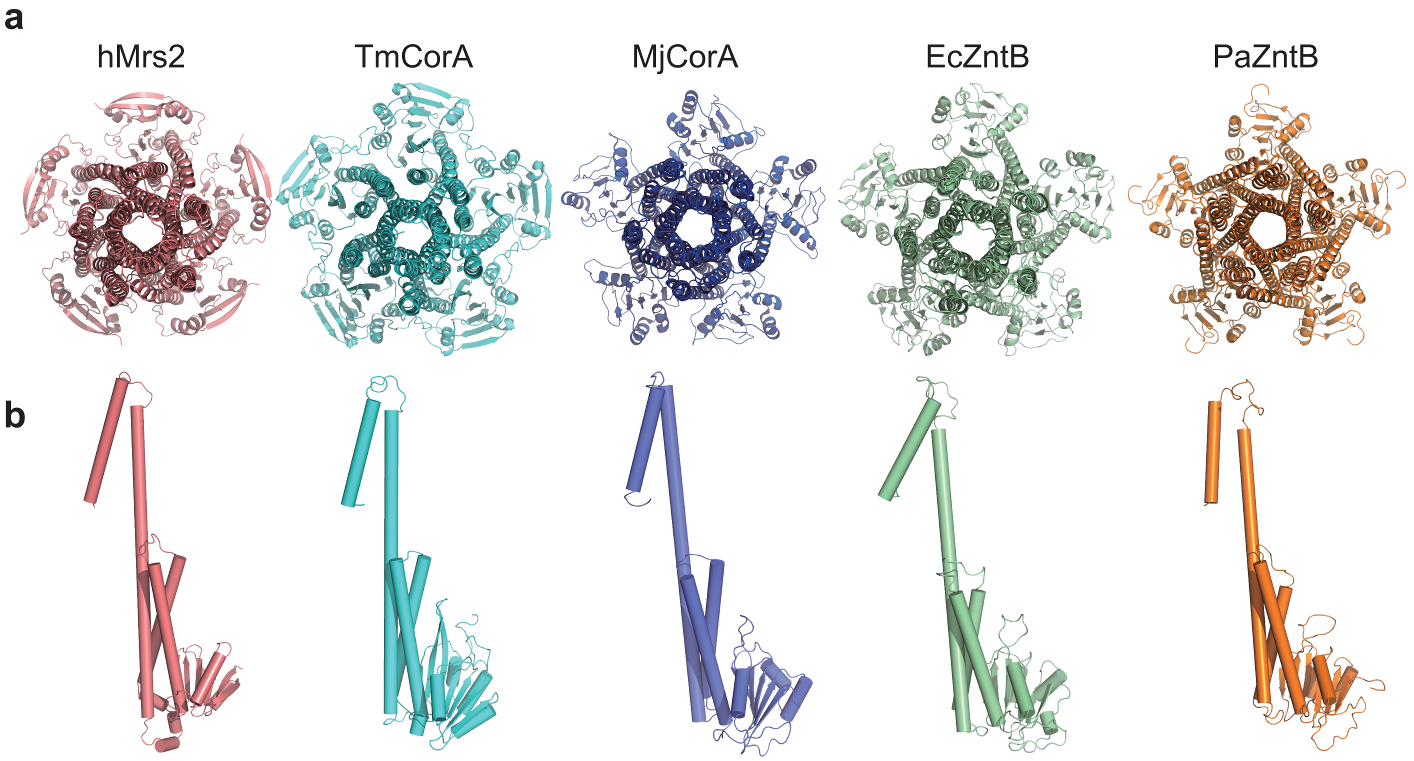

c

```
               1       10        20        30        40        50        60        70
Q9HD23|hMrs2   MECLRSLPCLLPRAMRLPRRTLCALALDVTSVGPPVAACGRRANLIGRSRAAQLCGPDRLRVAGEVHRFR
Q9WZ31|TmCorA  MEEKRLS......................AKKGLPPGTLVY....TGKYREDFEIEVMNYS.IEEFREFK
Q58439|MjCorA  MITVIAI......................AK....DGSIV..............EPKLDEIS.FED....
P64423|EcZntB  MEAIKG.......................SDVNVPDAVFAW....MLDGRG.............GVKPLE
G3XD00|PaZntB  MQAYES.........................GDERGLIYGY....VLNGRG.............GGRRVG

                                            80        90       100       110
Q9HD23|hMrs2   TSDVSQAT.....................LASVAPVFTVTKFDKQGNVTSFERKKTELYQELGLQARDLR
Q9WZ31|TmCorA  TTDVESVLPFRDSSTPTWINITGIHRTDVVQRVGEFFGIHPLVLEDILNVHQRPKVEFFENYVF......
Q58439|MjCorA  .............YRLIWIDCYDPKDEE.LYKLSKKIGISVSDLQIGLDEQEIPRVEEDEDFYL......
P64423|EcZntB  NTDV......IDEAHPCWLHLNYVHHDS.AQWLATTPLLPNNVRDALAGESTRPRVSRLGEGTL......
G3XD00|PaZntB  RNQIA..VLDLLPEESLWLHWDRGVPEA.QAWLRDSAGLSEFACDLLLEEATRPRLLDLGAESLL.....

             120       130       140       150       160          170        180
Q9HD23|hMrs2   FQHVMSITVRNNRIIMRMEYLKAVITPECLLILDYRNLNLE...QWLFRELPSQL.SGEGQLVTYPLPFE
Q9WZ31|TmCorA  .IVLKMFTYDKNLHELESEQVSLILTKNCVLMFQ.......EKIGDVFDPVRERIRYNRGI.......IR
Q58439|MjCorA  .IIYKAPLFEEDIT...TTSLGIYIKNNLLLTIHSDKIKAIGRLHKLISTKKPRIVF.............
P64423|EcZntB  .ITLRCINGSTDERPDQLVAMRVYMDGRLIVSTRQRKVLAL...DDVVSDLEE....G..........TG
G3XD00|PaZntB  .VFLRGVNLNPGAEPEDMVSLRVFADARRVISLRLRPLKAV...ADLLEDLEA....G..........KG

                 190       200       210       220            230       240       250
Q9HD23|hMrs2   FRAIEALLQYWINTLQGKLSILQPLILETLDALVDPK....HSSVDRSKLHILLQNGKSLSELETDIKIF
Q9WZ31|TmCorA  KKRADYLLYSLIDALVDDYFVLLEKIDDEIDVLEEEVLERPEKET.VQRTHQLK...RNLVEL.......
Q58439|MjCorA  ERGIGFLLYHILNEITRSYSRILMNLEDELEELEDKLLAGYDREV.MEKILGLR...KTLVYF.......
P64423|EcZntB  PTDCGGWLVDVCDALTDHSSEFIEQLHDKIIDLEDNL..LDQQIPPRGFLALLR...KQLIVM.......
G3XD00|PaZntB  PKTASEVVYYLAHYLTDRVDTLISGIADQLDAVEELVEADERASPDQHQLRTLR...RRSAGL.......

                      260       270       280             290       300       310
Q9HD23|hMrs2   KESILEILDEEELLEELCVSKWSDPQVFEKSS...A...GIDHAEEMELLLENYYRLADDLSNAARELRV
Q9WZ31|TmCorA  .................RKTIWPLREVLSSLYRDVPPLIEKETVPYFRDVYDHTIQIADTVETFRDIVSG
Q58439|MjCorA  .................HKSLIANRDVLVLLKRKYLPITTKEDRENFEDLYYDTLQLIDMSATYREVLTS
P64423|EcZntB  .................RRYMAPQRDVYARLASERLPWMSDDQRRRMQDIADRLGRGLDEIDACIARTGV
G3XD00|PaZntB  .................RRYLAPQRDIYSQLARYKLSWFVEDDADYWNELNNRLTRNLEELELIRERISV

                  320       330       340       350       360        370       380
Q9HD23|hMrs2   LIDDSQSIIFINLDSHRNVMMRLNLQLTMGTFSLSLFGLMGVAFGMNLESSLEE.DHRIFWLITGIMFMG
Q9WZ31|TmCorA  LLDVYLSSVSNKTNEVMKVLTIIATIFMPLTFIAGIY.......GMNFEYMPELRWKWGYPVVLAVM...
Q58439|MjCorA  MMDITLSLENIKMNQIMKILTMVTTIFAVPMWITGIY.......GMNFSYLPLANNPQGFWLVMALM...
P64423|EcZntB  MADEIAQVMQENLARRTYTMSLMAMVFLPSTFLTGLF.......GVNLGGIPGGGWQFGFSIFCILLVVL
G3XD00|PaZntB  LQEAESRRITERMNRTMYLLGIITGFFLPMSFVTGLL.......GINVGGIPGADAPHGFWLACLLI...

                         390       400       410       420       430       440
Q9HD23|hMrs2   SGLI.....W.RRLLSFLGRQLEAPLPPMMASLPKKTLLADRSMELKNSLRLDGLGSGRSSILTNR
Q9WZ31|TmCorA  .GVIAVIMVVYFKKKKWL..........................................ORA...
Q58439|MjCorA  .VVIIMIFVYIFRRSGWI..........................................ORA...
P64423|EcZntB  IGGVAL...W.LHRSKWL..........................................NTB...
G3XD00|PaZntB  .GGVATFQWWVFRRLRWL..........................................NTB...
```

Supplementary Fig. 5 Structure and sequence comparison of hMrs2, TmCorA, MjCorA, EcZntB and PaZntB. a Side-by-side pentamer comparison of hMrs2 (RCSB 8IP3), TmCorA (RCSB 4I0U), MjCorA (RCSB 4EV6), EcZntB (RCSB 5N9Y) and PaZntB (RCSB 7NH9) from the side view. **b** Side-by-side protomer comparison of hMrs2, TmCorA, MjCorA, EcZntB and PaZntB from the side view. **c** Sequence alignment among hMrs2 (UniProt ID: Q9HD23), TmCorA (UniProt ID: Q9WZ31), MjCorA (UniProt ID: Q58439), EcZntB (UniProt ID: P64423) and PaZntB (UniProt ID: G3XD00). The sequence number of hMrs2 is shown.

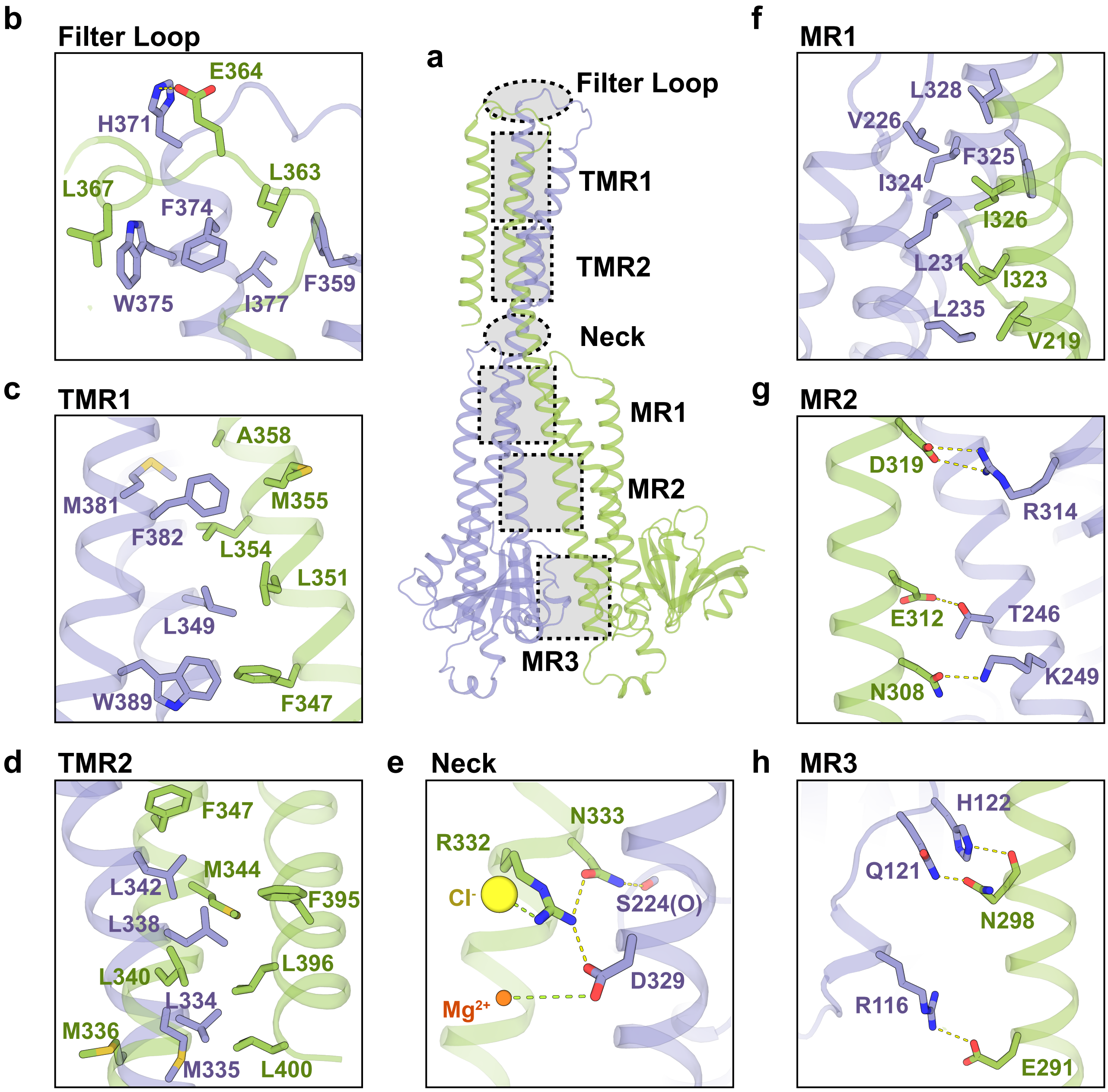

Supplementary Fig. 6 Protomer interactions. a Side view of the overall illustration showing the interactions between protomers. The two protomers are shown in blue and green. **b-h** Details of the interaction of the Filter Loop (**b**), TMR1 (**c**), TMR2 (**d**), Neck (**e**), MR1 (**f**), MR2 (**g**) and MR3 (**h**). The residue side chains involved in the interaction are shown as sticks. Hydrogen bonds are represented by dashed lines. The color of carbon atoms depends on the protomer from which they were drived. Nitrogen, oxygen, magnesium and chloride atoms are colored blue, red, orange and yellow, respectively. All interactions shown in (**b**)-(**h**) occur in every pair of adjacent protomers in the pentamer.

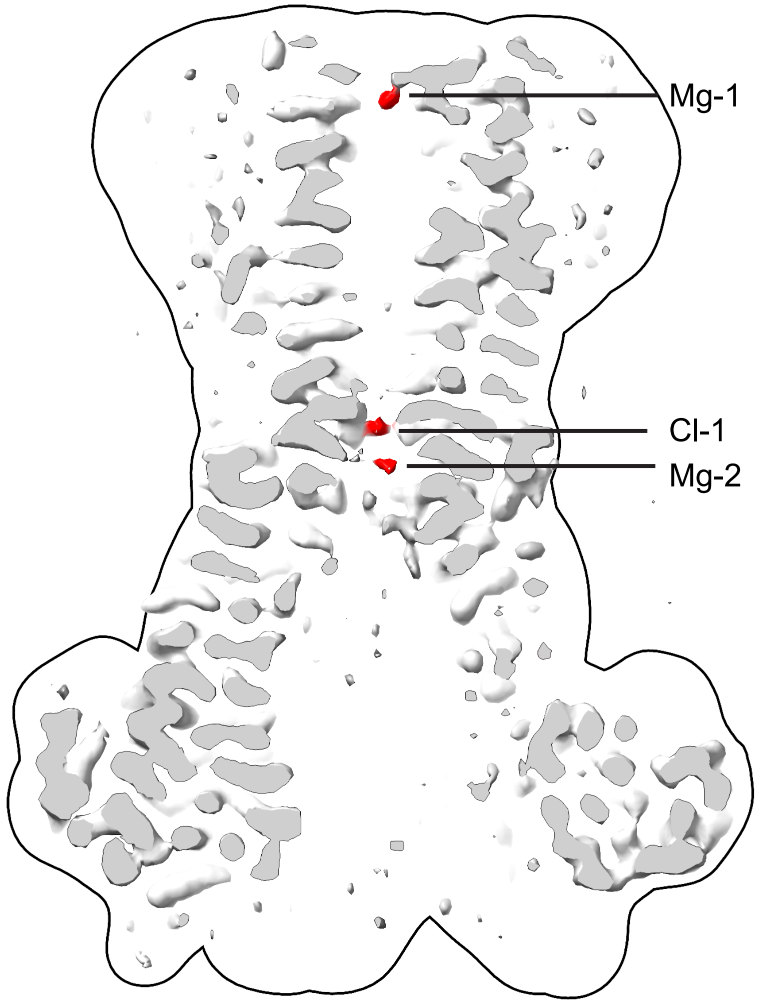

Supplementary Fig. 7 Cryo-EM reconstruction cross-section of hMrs2-highEDTA with C1 symmetry. The density maps of the ion binding sites (Mg-1, Cl-1 and Mg-2) are red, and the density maps of hMrs2-highEDTA are gray.

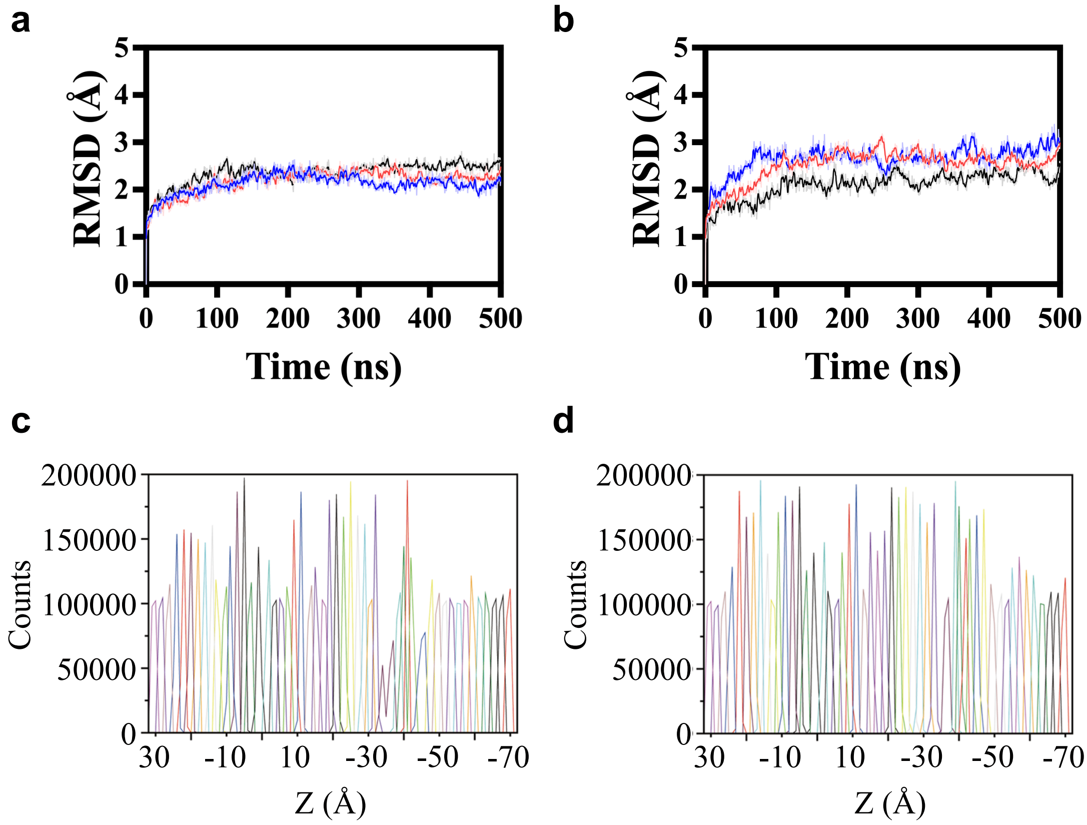

Supplementary Fig. 8 The assessment of MD simulations system and PMF calculation convergence in Mrs2-withCl$^-$ and Mrs2-noCl$^-$ system. a-b The time-course RMSD values for the Cα atoms of the Mrs2-withCl$^-$ or Mrs2-noCl$^-$ structure relative to the cryo-EM structure in three independent Su-GaMD simulations for Mrs2-withCl$^-$ (**a**) and Mrs2-noCl$^-$ (**b**) system. **c-d** The umbrella histogram of Mg^{2+} Z-position (**c**) with or (**d**) without Cl$^-$ shows good spacing and overlap along all reaction coordinates.

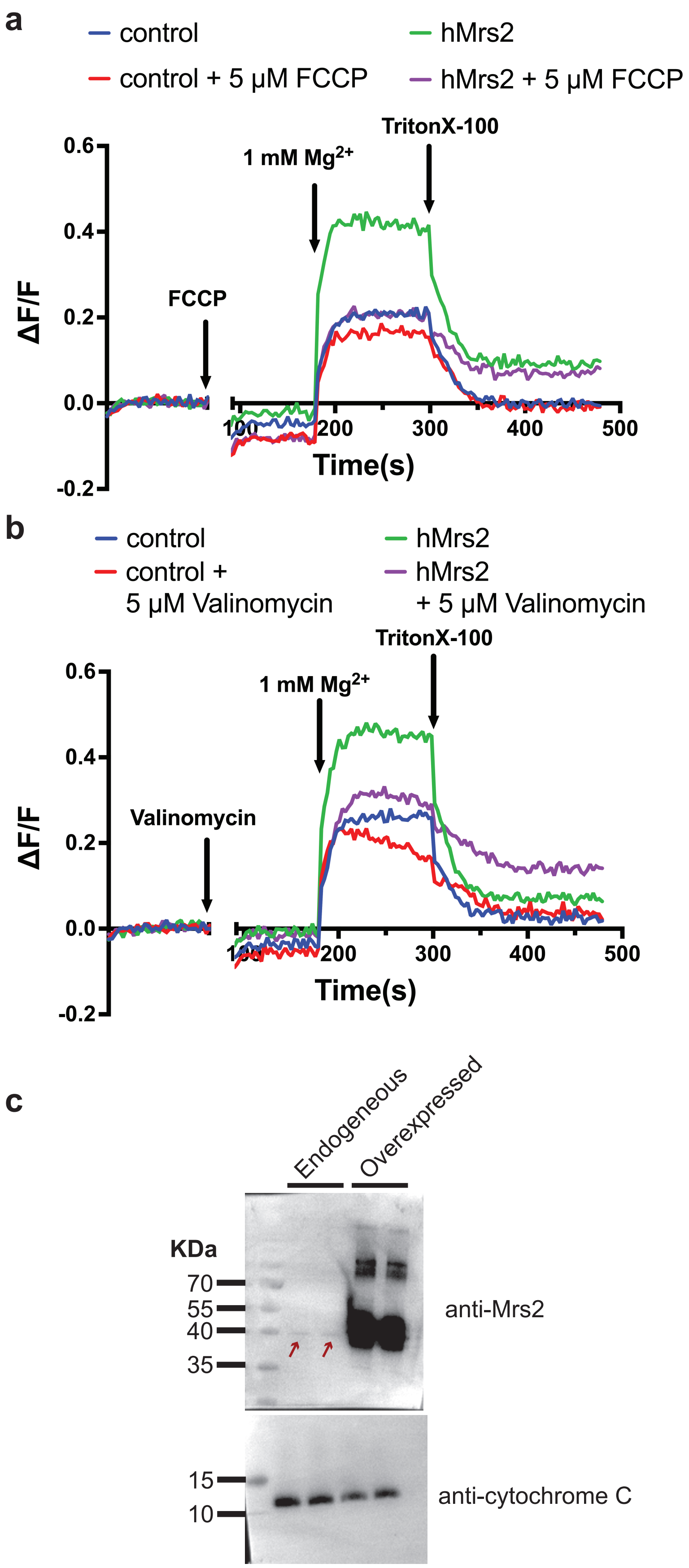

Supplementary Fig. 9 Mitochondrial Mg^{2+} uptake by hMrs2 depends on the membrane potential. a-b Representative raw profiles of the mitochondrial Mg^{2+} uptake assay with purified mitochondria overexpressing hMrs2 after treatment with FCCP (**a**) or valinomycin (**b**). Mitochondria with endogenous hMrs2 served as controls. **c** Representative western blots of purified mitochondria used in the assay to measure endogenous hMrs2 or overexpressed hMrs2 protein. Red arrows indicate bands of endogenous hMrs2. Rabbit anti-MRS2 antibody (Sigma-Aldrich, HPA017642, 1:1000) and mouse anti-cytochrome C (Abcam; ab110325, 1:2000) were used. This experiment was repeated independently with similar results three times.

Supplementary Table 1

Cryo-EM data collection, refinement and validation statistics

	hMrs2-Mg	hMrs2-lowEDTA	hMrs2-highEDTA	hMrs2-rest
**Data Collection and processing**				
Microscopy	Titan Krios			
Detector	Falcon 4	Falcon 4	Falcon 4	K2
Magnification	130,000	130,000	130,000	290,000
Voltage (kV)	300			
Electron exposure ($e^-/Å^2$)	59.97	59.97	51.39	54.0
Defocus range (μm)	-0.8 to -1.6	-0.8 to -1.6	-0.8 to -1.6	-0.8 to -1.6
Pixel size (Å)	0.93	0.93	0.93	1.014
Micrographs collected	3,329	3,217	1,708	695
Total extracted particles	473,653	631,480	374,846	170,283
Final particle number	304,325	185,000	171,385	104,000
(Symmetry expanded by C5)				
Symmetry of final map	*C1*	*C1*	*C1*	*C1*
Map resolution	2.6	2.5	2.7	2.9
FSC threshold	0.143	0.143	0.143	0.143
Map sharpening B factor ($Å^2$)	66.7	57.9	61.5	51.4
**Model composition**				
Nonhydrogen atoms	12,868	12,928	12,933	12,921
Protein residues	1,595	1,600	1,600	1,600
Ligands	13	3	3	1
Water	0	5	5	0
**Refinement**				
Refinement package	PHENIX			
Model resolution (Å)	2.7	2.7	2.9	3.1
FSC threshold	0.5	0.5	0.5	0.5
B factor protein ($Å^2$)	83.03	88.37	114.77	93.92
B factor ligand ($Å^2$)	83.08	75.23	103.91	76.58
B factor water ($Å^2$)	-	75.50	92.83	-
R.m.s.d. bond lengths (Å)	0.002	0.002	0.002	0.002
R.m.s.d. bond angles (°)	0.425	0.434	0.442	0.427
**Validation**				
MolProbity score	1.29	1.35	1.19	1.12
Clash score	5.46	6.27	4.04	2.5
Rotamer outliers (%)	0	0	0	0
Ramachandran favored (%)	98.11	98.43	98.43	97.61
Ramachandran allowed (%)	1.89	1.57	1.57	2.39
Ramachandran disallowed (%)	0	0	0	0

Supplementary Table 2

An overview of the MD system presented in this study

**System**	Box dimensions (Å)	Total number of atoms	Total number of water molecules	Salt concentration	lipid
Mrs2-withCl^-	135 × 135 × 180	316,626	75,402	0.15 M NaCl, 0.02 M $MgCl_2$	POPC
Mrs2-noCl^-	135 × 135 × 180	316,624	75,402	0.15 M NaCl, 0.02 M $MgCl_2$	POPC

Supplementary Table 3

Non-bonded interaction parameters of cations

	σ_i	ε_i	q_i
Na^+	0.3810	0.00061	1.000
Mg^{2+}	0.1630	0.59000	2.000
