# Supplementary material for: Molecular basis of Mg2+ permeation through the human mitochondrial Mrs2 channel
Source: Nat Commun. 2023 Aug 5;14:4713. doi: 10.1038/s41467-023-40516-2 (PMC10404273; doi:10.1038/s41467-023-40516-2)
Supplement: Supplementary file 3 — Description of Additional Supplementary Files [file 41467_2023_40516_MOESM3_ESM.pdf]

Description of Additional Supplementary Files

File Name: Supplementary Data 1
Description: Primers sequence
