# Supplementary material for: Molecular basis of Mg2+ permeation through the human mitochondrial Mrs2 channel
Source: Nat Commun. 2023 Aug 5;14:4713. doi: 10.1038/s41467-023-40516-2 (PMC10404273; doi:10.1038/s41467-023-40516-2)
Supplement: Supplementary file 5 — Reporting Summary [file 41467_2023_40516_MOESM5_ESM.pdf]

nature portfolio

Corresponding author(s): Xue Yang, Yuequan Shen

Last updated by author(s): Jul 18, 2023

Reporting Summary

Nature Portfolio wishes to improve the reproducibility of the work that we publish. This form provides structure for consistency and transparency in reporting. For further information on Nature Portfolio policies, see our Editorial Policies and the Editorial Policy Checklist.

Statistics

For all statistical analyses, confirm that the following items are present in the figure legend, table legend, main text, or Methods section.

n/a	Confirmed	
☐	☒	The exact sample size (*n*) for each experimental group/condition, given as a discrete number and unit of measurement
☐	☒	A statement on whether measurements were taken from distinct samples or whether the same sample was measured repeatedly
☐	☒	The statistical test(s) used AND whether they are one- or two-sided<br>*Only common tests should be described solely by name; describe more complex techniques in the Methods section.*
☒	☐	A description of all covariates tested
☐	☒	A description of any assumptions or corrections, such as tests of normality and adjustment for multiple comparisons
☐	☒	A full description of the statistical parameters including central tendency (e.g. means) or other basic estimates (e.g. regression coefficient) AND variation (e.g. standard deviation) or associated estimates of uncertainty (e.g. confidence intervals)
☐	☒	For null hypothesis testing, the test statistic (e.g. *F*, *t*, *r*) with confidence intervals, effect sizes, degrees of freedom and *P* value noted<br>*Give P values as exact values whenever suitable.*
☒	☐	For Bayesian analysis, information on the choice of priors and Markov chain Monte Carlo settings
☒	☐	For hierarchical and complex designs, identification of the appropriate level for tests and full reporting of outcomes
☒	☐	Estimates of effect sizes (e.g. Cohen's *d*, Pearson's *r*), indicating how they were calculated

Our web collection on statistics for biologists contains articles on many of the points above.

Software and code

Policy information about availability of computer code

Data collection	SerialEM software (3.8), EPU software (2.14), AMBER-2020
Data analysis	MotionCor2 software (1.3.2), CTFFind software (4.0.8), Gautomatch software (0.56), RELION software (3.1 or 4), CryoSPARC software (3.3.2), PHENIX software (1.19), Coot software (0.8.9.3), Pymol software (2.2.0), UCSF ChimeraX software (1.5), VMD software (1.9.3), GraphPad Prism (9.1.1), HOLE software (2.2.005), ResMap software (1.1.4), H++ program, SHAKE algorithm, PME algorithm, WHAM software (2.0.11)

For manuscripts utilizing custom algorithms or software that are central to the research but not yet described in published literature, software must be made available to editors and reviewers. We strongly encourage code deposition in a community repository (e.g. GitHub). See the Nature Portfolio guidelines for submitting code & software for further information.

Data

Policy information about availability of data

All manuscripts must include a data availability statement. This statement should provide the following information, where applicable:

- Accession codes, unique identifiers, or web links for publicly available datasets
- A description of any restrictions on data availability
- For clinical datasets or third party data, please ensure that the statement adheres to our policy

The data that support this study are available in a publicly accessible repository. Atomic coordinates have been deposited in the Protein Data Bank under accession number 8IP3, 8IP6, 8IP5 and 8IP4 for hMrs2-Mg, hMrs2-rest, hMrs2-lowEDTA and hMrs2-highEDTA, respectively. Cryo-EM density maps have been deposited in the Electron Microscopy Data Bank under accession number EMD-35630, EMD-35633, EMD-35632 and EMD-35631 for hMrs2-Mg, hMrs2-rest, hMrs2-lowEDTA and

hMrs2-highEDTA, respectively. Alphafold2 database, PDB code: 4I0U, 4EV6, 5N9Y, 7NH9 , UniProt ID: Q9HD23, Q9WZ31, Q58439, P64423, G3XD00 were used in this study. The MD simulations data have been deposited to Zenodo (https://doi.org/10.5281/zenodo.8153491). Source data are provided with this paper.

Research involving human participants, their data, or biological material

Policy information about studies with human participants or human data. See also policy information about sex, gender (identity/presentation), and sexual orientation and race, ethnicity and racism.

Reporting on sex and gender	N/A
Reporting on race, ethnicity, or other socially relevant groupings	N/A
Population characteristics	N/A
Recruitment	N/A
Ethics oversight	N/A

Note that full information on the approval of the study protocol must also be provided in the manuscript.

Field-specific reporting

Please select the one below that is the best fit for your research. If you are not sure, read the appropriate sections before making your selection.

[x] Life sciences [ ] Behavioural & social sciences [ ] Ecological, evolutionary & environmental sciences

For a reference copy of the document with all sections, see nature.com/documents/nr-reporting-summary-flat.pdf

Life sciences study design

All studies must disclose on these points even when the disclosure is negative.

Sample size	For cryo-EM data collection, a total of 690 image stacks were collected for hMrs2-rest sample by a 300 kV Titan Krios G3 cryo-electron microscope (FEI) equipped with K2 Summit direct electron detector (Gatan); 3,329 image stacks of hMrs2-Mg, 3,217 image stacks of hMrs2-lowEDTA and 1,708 image stacks of hMrs2-highEDTA were collected by a 300 kV Titan Krios G3 cryo-electron microscope (FEI) equipped with Falcon4 direct electron detector and Selectris energy filter. For mitochondrial Mg2+ uptake assay, no sample size predetermination was performed. The reported sample size was based on published works and was sufficient to obtain reproducible and reliable data for analysis. Amount of MD data collected was limited by computational resources available.
Data exclusions	No any data was systematically excluded. Poor quality images (ice contamination, empty carbon, distorted) were removed manually. Particles in bad classes were discarded and the data processing flowchart were summarized in Supplementary Figures. These criteria were established in softwares (RELION & CryoSPARC) for cryo-EM image analysis.
Replication	The numbers of replication were described in related legends. Experimental findings were reliably reproduced.
Randomization	For EM single particle analysis and MD simulations, samples were allocated into experimental groups randomly. Randomization is not relevant to the mitochondrial Mg2+ uptake assays, as all data were used in the analysis.
Blinding	Blinding is unnecessary or invalid for the purpose of structure determination, mitochondrial Mg2+ uptake assays and MD simulations.

Reporting for specific materials, systems and methods

We require information from authors about some types of materials, experimental systems and methods used in many studies. Here, indicate whether each material, system or method listed is relevant to your study. If you are not sure if a list item applies to your research, read the appropriate section before selecting a response.

Materials & experimental systems

n/a	Involved in the study	
[ ]	[x]	Antibodies
[ ]	[x]	Eukaryotic cell lines
[x]	[ ]	Palaeontology and archaeology
[x]	[ ]	Animals and other organisms
[x]	[ ]	Clinical data
[x]	[ ]	Dual use research of concern
[x]	[ ]	Plants

Methods

n/a	Involved in the study	
[x]	[ ]	ChIP-seq
[x]	[ ]	Flow cytometry
[x]	[ ]	MRI-based neuroimaging

Antibodies

Antibodies used	The following antibodies were used. Primary antibodies: rabbit anti-MRS2 antibody (Sigma-Aldrich, HPA017642, 1:1000), mouse anti-Cytochrome C (Abcam; ab110325, 1:2000); Secondary antibodies: rabbit anti-Mouse IgG (Abcam, ab6728, 1:8000), goat anti-Rabbit (Abcam, ab6721, 1:2000)
Validation	Validation statement was available at the web page for each antibody:<br>Rabbit anti-MRS2 antibody: https://www.sigmaaldrich.cn/CN/zh/product/sigma/hpa017642<br>Mouse anti-Cytochrome C: https://www.abcam.com/products/primary-antibodies/cytochrome-c-antibody-37ba11-ab110325.html

Eukaryotic cell lines

Policy information about cell lines and Sex and Gender in Research

Cell line source(s)	HEK293, ATCC, Cat#CRL-1573
Authentication	No additional authentication was performed by the authors of this study.
Mycoplasma contamination	the cell line in this study was tested negative for mycoplasma contamination.
Commonly misidentified lines<br>(See ICLAC register)	No commonly misidentified cell lines were used.
